# Heterogeneity among *Mycobacterium avium* complex species isolated from pulmonary infection in Taiwan

**DOI:** 10.1128/spectrum.00309-25

**Published:** 2025-07-07

**Authors:** Hsiu-Mei Lin, Chin-Chung Shu, Chun-Hao Chen, Nan-Yu Chen, Jeng-How Yang, Chih-Hung Chen, Shih-Hong Li, Chih-Liang Wang, Chih-Teng Yu, Shu-Min Lin, Kuo-Chin Kao, Chung-Chi Huang, Cheng-Ta Yang, Jang-Jih Lu, Cheng-Hsun Chiu, Hsin-Chih Lai, Ting-Shu Wu

**Affiliations:** 1 Department of Family Medicine, Linkou Chang Gung Memorial Hospital 38014, Taoyuan, Taiwan; 2 Department of Internal Medicine, National Taiwan University Hospital, National Taiwan University School of Medicine 38006, , Taipei, Taiwan; 3 Department of Orthopedics, Chiayi Chang Gung Memorial Hospital 125573, , Chiayi, Taiwan; 4 Division of Infectious Diseases, Department of Internal Medicine, Linkou Chang Gung Memorial Hospital 38014, Taoyuan, Taiwan; 5 School of Medicine, Chang Gung University 520657, Taoyuan, Taiwan; 6 Division of Infectious Diseases, Department of Internal Medicine, New Taipei Municipal TuCheng Hospital (Built and Operated by Chang Gung), New Taipei City, Taiwan; 7 Division of Thoracic Medicine, Department of Internal Medicine, Linkou Chang Gung Memorial Hospital 38014, Taoyuan, Taiwan; 8 Department of Respiratory Therapy, Chang Gung University, Taoyuan, Taiwan; 9 Division of Clinical Pathology, Taipei Tzu Chi Hospital, Buddhist Tzu Chi Medical Foundation, New Taipei City, Taiwan; 10 Department of Pediatrics, Linkou Chang Gung Memorial Hospital, Taoyuan, Taiwan; 11 Department of Medical Technology and Laboratory Science, College of Medicine, Chang Gung University 71589, Taoyuan, Taiwan; Taichung Veterans General Hospital, Taichung, Taiwan

**Keywords:** concatenated gene sequences, phylogenetic analysis, multidrug resistance, genetic diversity, combination therapy

## Abstract

*Mycobacterium avium* complex (MAC) is an emerging pathogen causing nontuberculous pulmonary infections globally. However, clinical treatment guidelines regard MAC as a single entity, recommending a universal anti-mycobacterial combination therapy. Our study aimed to distinguish species among MAC and investigate the antimicrobial susceptibility for the selection of optimal antimicrobial agents in Taiwan. Two hundred ninety-four consecutive sputum samples confirmed as MAC by matrix-assisted laser desorption/ionization time-of-flight mass spectrometry were collected from 1 November 2015 to 31 August 2020 at Linkou Chang Gung Memorial Hospital in Taiwan. These isolates were identified through 16S rRNA gene, 23S rRNA gene, heat-shock protein 65 gene (*hsp65*), internal transcribed spacer, and beta subunit of RNA polymerase (*rpoB*) gene sequencing and phylogenetic analyses. Antimicrobial susceptibility testing (AST) was performed with 13 antimicrobial agents. The predominant pathogen identified was *Mycobacterium intracellulare* clade A (122/294, 41.5%), followed by *M. intracellulare* subsp. *chimaera* (87/294, 29.6%), *M. intracellulare* subsp. *intracellulare* (39/294, 13.3%), *M. avium* (35/294, 11.9%), and four other species (11/294, 3.7%). AST showed that clarithromycin and amikacin had high susceptibility rates against *M. intracellulare* clade A, *M. intracellulare* subsp. *chimaera*, *M. intracellulare* subsp. *intracellulare,* and *M. avium*, while linezolid and moxifloxacin exhibited higher resistance. The comparison of minimum inhibitory concentrations among species within the same antimicrobial agent showed variability in susceptibility. A diverse clonality of *M. intracellulare* might exist in MAC pulmonary infections in Taiwan. Among MAC species, *M. avium* exhibited multidrug resistance. Although international guidelines recommend macrolide, ethambutol, and rifampin for treating MAC pulmonary disease, our study highlights the importance of considering species identification and regional AST results when selecting anti-mycobacterial agents.

**IMPORTANCE:**

There are more than 10 (sub)species within the *Mycobacterium avium* complex. According to modern biotechnology, such as matrix-assisted laser desorption/ionization time-of-flight mass spectrometry, it is still difficult to differentiate the complex into specific species precisely. We utilize concatenated multi-gene sequencing to classify this complex at the (sub)species level. Indeed, we encountered poorer treatment outcomes when facing *Mycobacterium avium* pulmonary infections compared to other species causing pulmonary infections. Individualized anti-mycobacterial therapy should focus on each species responsible for pulmonary disease.

## INTRODUCTION

*Mycobacterium avium* complex (MAC) belongs to the slowly growing mycobacteria, which are ubiquitous in the environment (1). It causes pulmonary infection, skin and soft tissue infection, bloodstream infection, bone and joint infection, and disseminated infection in not only immunocompetent but also immunocompromised hosts (2, 3). It is the leading causative agent of nontuberculous pulmonary infection in the world.

In addition to its pathogenicity, the taxonomy of MAC is mysterious. According to the study by van Ingen et al*.* (4)*,* MAC is composed of 12 species: *Mycobacterium avium*, *Mycobacterium intracellulare*, *Mycobacterium chimaera*, *Mycobacterium colombiense*, *Mycobacterium arosiense*, *Mycobacterium vulneris*, *Mycobacterium bouchedurhonense*, *Mycobacterium timonense*, *Mycobacterium marseillense*, *Mycobacterium yongonense*, *Mycobacterium paraintracellulare,* and *Mycobacterium lepraemurium*.

Conventional biochemical methods fail to identify species among the MAC precisely. Even modern technology, such as matrix-assisted laser desorption/ionization time-of-flight mass spectrometry (MALDI-TOF/MS), does not easily distinguish the species precisely. For example, *M. intracellulare* subsp. *intracellulare* may reveal the same mass-to-charge (*m/z*) pattern with *M. intracellulare* subsp. *chimaera* (5).

Concatenated multiple gene sequencing can be a valuable alternative to whole-genome sequencing (WGS) to overcome the limitations of conventional biochemical methods and MALDI-TOF/MS (4, 6). Any housekeeping or ribosomal gene can be used for gene expression studies in the most validated species (4). The 16S rRNA gene, as the diagnosis of choice, is commonly used in bacterial identification studies. However, it has high sequence similarity among different species, which is not suitable for analyzing closely related species. Thus, consideration should be given to utilizing other housekeeping or ribosomal gene sequencing to distinguish closely related species. Both the heat-shock protein 65 gene (*hsp65*) and the beta subunit of RNA polymerase (*rpoB*) gene are effective and reliable in identifying phylogenetic relationships within the genus *Mycobacterium* (7–9). 16S-23S rRNA gene internal transcribed spacer (ITS) is also useful to distinguish the species among the *Mycobacterium* genus that is often misidentified by the 16S rRNA gene alone (10).

Furthermore, though we know the MAC is composed of already known 12 species, according to current treatment guidelines of the MAC published by the American Thoracic Society, European Respiratory Society, European Society of Clinical Microbiology and Infectious Diseases and Infectious Diseases Society of America (ATS/ERS/ESCMID/IDSA), the pulmonary diseases caused by MAC are recommended to be treated by a universal anti-mycobacterial combination therapy regimen, which considered the MAC as a single entity without specific species within the complex (11).

The goals of this study are to identify the precise species of MAC isolates in Taiwan and explore the antimicrobial susceptibility patterns among each species, which may thereby help us to select the most appropriate anti-mycobacterial agent(s) for individual species of MAC-associated infection.

## MATERIALS AND METHODS

### Sequence analysis of clinical isolates

#### Clinical entities of MAC pulmonary infection cases

A total of 294 consecutive and non-repeated sputum isolates that were confirmed as MAC by MALDI-TOF/MS were collected from 294 consecutive patients at Linkou Chang Gung Memorial Hospital in Taiwan between 1 November 2015 and 31 August 2020. These isolates were stored in skim milk with 50% glycerol at –70°C until they were subcultivated. Among these 294 patients, 2 were coinfected with *Mycobacterium abscessus* complex, 1 was coinfected with *Mycobacterium fortuitum*, 2 were coinfected with *Mycobacterium gordonae*, 1 was coinfected with *Mycobacterium kansasii*, and 3 were coinfected with *Mycobacterium tuberculosis* complex.

#### Matrix-assisted laser desorption/ionization time-of-flight mass spectrometry identification

The extraction protocol followed the manufacturer’s instructions. A volume of 300 µL of ultrapure water was pipetted into a 1.5 mL Eppendorf Safe-Lock tube. One to three 10 µL inoculation loops of Mycobacteria biomass were transferred into the tube. To get an idea of the amount of biomass required: 2 μL water in an Eppendorf tube represents a small pellet, 5 μL of water represents a suitably sized pellet. Heat inactivation was performed by boiling for 30 min. After cooling, 900 µL of 70% ethanol was pipetted into the tube and mixed using a vortex mixer. The mixture was centrifuged at maximum speed (≥13,000 rpm) for 2 min, and the supernatant was decanted. It was centrifuged again to remove any remaining ethanol by pipetting. The pellet was allowed to dry at room temperature for a few minutes.

A small spatula tip of zirconia/silica beads was added to the tube, followed by 10–50 µL of pure acetonitrile, depending on the pellet size. The tube was then vortexed at maximum speed for 1 min. An equal volume of 70% formic acid was added, mixed for 5 seconds, and centrifuged again at ≥13,000 rpm for 2 min. One microliter of the supernatant was placed on a MALDI target plate and allowed to dry at room temperature. Subsequently, 1.0 µL of α-cyano-4-hydroxycinnamic acid solution was overlaid onto the dried sample and allowed to dry again. In our hospital, the MALDI-TOF/MS system used was a Biotyper Microflex LT/SH (Bruker Daltonics), and the software was MALDI Biotyper version 3.1 with the Mycobacteria Library version 2.0. Spectra were obtained in positive linear mode over a mass-to-charge (*m/z*) ratio of 2,000–20,000 Da, with an accelerating voltage of 20 kV. Measurements were conducted automatically using a nitrogen laser at 40 shots per second, collecting 240 laser shots per spot. The log(score) cutoffs recommended by the manufacturer were <1.6999, identification not reliable; 1.700–1.999, probable genus identification; 2.000–2.229, secure genus identification and probable species identification; and 2.3000–3.000, highly probable species identification.

#### DNA extraction and gene sequencing of 16S rRNA, 23S rRNA, *hsp65*, ITS, and *rpoB*

Two hundred and ninety-four strains of bacterial genomic DNA were extracted by the High Pure Viral Nucleic Acid Kit (Roche, Mannheim, Germany) according to the manufacturer’s protocol. Subspeciation of the 294 MAC isolates was based on sequences of the five housekeeping genes: 16S rRNA, 23S rRNA, *hsp65*, ITS, and *rpoB*. The details of the polymerase chain reaction method were described previously by Zelazny et al. (12) and Jong et al. (13). The sequencing method used is the Sanger method. The sequences of these five genes of each isolate were analyzed and compared with the NCBI database by using the BLAST method (https://blast.ncbi.nlm.nih.gov/Blast.cgi). The primers used and amplification conditions for DNA extraction are shown in Table 1.

**TABLE 1:** Primers and amplification conditions used for 16S rRNA, 23S rRNA, *hsp65*, ITS, and *rpoB* gene sequencing

Gene	Primer	Total length (bp)	Sequence	Amplification conditions	Reference
16S rRNA	pA	1,523–1,525	5′-AGAGTTTGATCCTGGCTCAG-3′	35 cycles of 95°C for 1 min; 62°C for 1 min; 72°C for 1 min.	(14)
	pEr		5′-CCGTCAATTCCTTTGAGTTT-3′		
	pD		5′-CAGCAGCCGCGGTAATAC-3′		
	pHr		5′-AAGGAGGTGATCCAGCCGCA-3′		
23S rRNA	19F	835–836	5′-GTAGCGAAATTCCTTGTCGG-3′	35 cycles of 95°C for 1 min; 60°C for 1 min; 72°C for 1 min.	(15)
	21R		5′-TTCCCGCTTAGATGCTTTCAG-3′		
*hsp65*	Tb11	441	5′-ACCAACGATGGTGTGTCCAT-3′	35 cycles of 95°C for 1 min; 60°C for 1 min; 72°C for 1 min.	(16)
	Tb12		5′-CTTGTCGAACCGCATACCCT-3′		
ITS	16S-1511f	368–371	5′-AAGTCGTAACAAGGTARCCG-3′	35 cycles of 95°C for 1 min; 64°C for 1 min; 72°C for 1 min.	(17)
	23S-23r		5′-TCGCCAAGGCATCCACC-3′		
*rpoB*	Myco F	752	5′-GGCAAGGTCACCCCGAAGGG-3′	35 cycles of 95°C for 1 min; 60°C for 1 min; 72°C for 1 min.	(7)
	Myco R		5′-AGCGGCTGCTGGGTGATCATC-3′		

### Phylogenetic analysis

16S rRNA, 23S rRNA, *hsp65*, ITS, and *rpoB* genes of MAC isolates were analyzed and compared with type strains in the NCBI database for phylogenetic trees by MEGA-XI software. The multisequence alignment, including full-length 16S rRNA (1,426 bp), partial *hsp65* (354 bp), and partial *rpoB* (680 bp) genes, was calculated by using the CLUSTAL W program, followed by the neighbor-joining method with Kimura’s two-parameter procedure to construct the phylogenetic tree and displayed as the circular pattern (18). All 5′ and 3′ ambiguous positions were removed for each sequence pair. We used 18 type strains, including *M. avium* subsp. *hominissuis* 104, *M. avium* subsp. *paratuberculosis* ATCC 19698^T^, *M. avium* subsp. *silvaticum* ATCC 49884^T^, *M. avium* subsp. *avium* ATCC 25291^T^, *M. intracellulare* ATCC 13950^T^, *M. intracellulare* MOTT-64, *M. intracellulare* subsp. *intracellulare* S-24, *M. intracellulare* subsp. *intracellulare* FDAARGOS 1610, *M. intracellulare* subsp. *chimaera* DSM 44623^T^, *M. intracellulare* subsp. *chimaera* CDC 2015-22-71, *M. yongonense* 05-1390^T^, *M. timonense* CIP 109830^T^, *M. marseillense* 5356591^T^, *M. colombiense* CECT 3035^T^, *M. arosiense* T1921^T^, *M. bouchedurhonense* CIP 109827^T^, *M. lepraemurium* str. Hawaii, and *M. vulneris* NLA000700772^T^.

### Antimicrobial susceptibility testing

Antimicrobial susceptibility testing (AST) was performed by using the broth microdilution methods with Sensititre SLOMYCO MIC plates (Thermo Fisher, Cleveland, OH, USA) recommended by the Clinical and Laboratory Standards Institute (CLSI) (19). The antimicrobial agents included amikacin (AMI), ciprofloxacin (CIP), clarithromycin (CLA), doxycycline (DOX), ethambutol (EMB), ethionamide (ETH), isoniazid (INH), linezolid (LZD), moxifloxacin (MXF), rifabutin (RFB), rifampin (RIF), streptomycin (STR), and trimethoprim/sulfamethoxazole (SXT). The MICs of the antibiotics mentioned above were read on the seventh day after subcultivation if the growth was enough. The MIC was defined as the lowest drug concentration that inhibits visible growth. The exception was SXT, for which the endpoint was the well showing approximately 80% growth inhibition compared with the growth in the control well with no drug. MIC_50_ and MIC_90_ were defined as the MIC to inhibit the growth of 50% and 90% of the isolates, respectively. The intermediate breakpoints (in μg/mL) of these antibiotics were those proposed by CLSI (20), with CLA = 16, AMI = 32, MXF = 2, and LZD = 16. The susceptibility categories for RIF were suggested by van Ingen et al. (21) as follows: susceptible (MICs of ≤0.5 µg/mL), intermediate (MICs of 1, 2, or 4 µg/mL), and resistant (MICs of ≥8 µg/mL). The MICs of *M. avium* ATCC 700898 were used as the quality control.

### Statistical analysis

Stata 17 (StataCorp LLC, College Station, TX, USA) and GraphPad Prism 9 were used as the statistical software to analyze the MICs, MIC_50_, and MIC_90_, as well as to perform multiple-group and two-group comparisons based on nonparametric theory. The Kruskal-Wallis test was employed for multiple-group comparisons to determine whether there were statistically significant differences among 13 antimicrobial agents (*P* value < 0.05). For further analysis between two species within these MAC species, the Mann-Whitney *U* test was used to calculate differences among the 13 antimicrobial agents.

## RESULTS

### Species identification of 294 isolates

**Fig 1:**
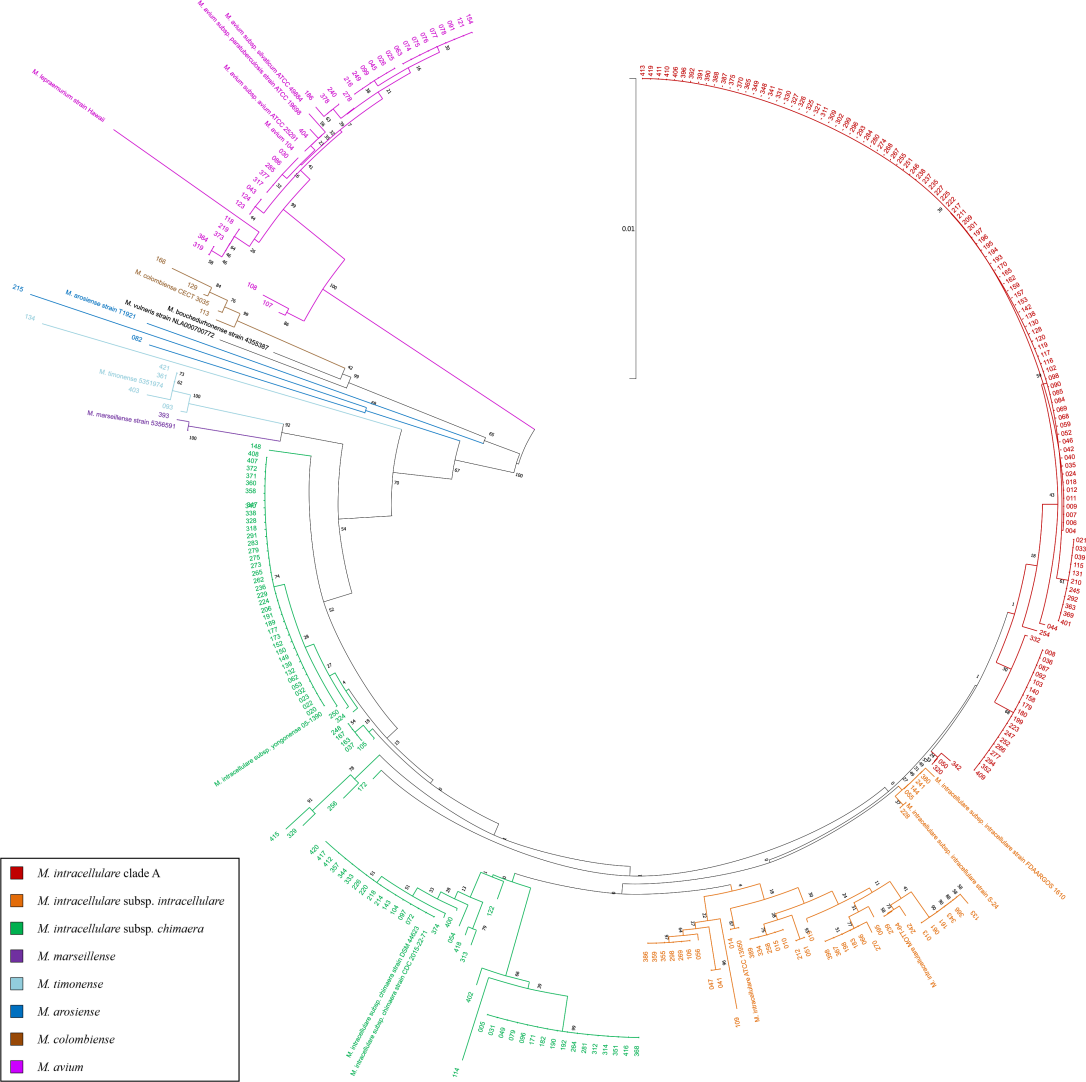
The phylogenetic tree of full-length 16S rRNA-partial *hsp65*-partial *rpoB* of 294 isolates and type strains. The multisequence alignment, including full-length 16S rRNA (1,426 bp), partial *hsp65* (354 bp), and partial *rpoB* (680 bp) genes, was calculated by using the CLUSTAL W program, followed by the neighbor-joining method with Kimura’s two-parameter procedure to construct the phylogenetic tree and displayed as the circular pattern (18).

In concatenated multiple gene phylogenetic analysis, including full-length 16S rRNA-partial *hsp65*-partial *rpoB* (Fig. 1), *M. intracellulare* clade A (122/294, 41.5%) comprised the majority, followed by *M. intracellulare* subsp. *chimaera* (87/294, 29.6%), *M. intracellulare* subsp. *intracellulare* (39/294, 13.3%), *M. avium* (35/294, 11.9%), and other species, which included *M. timonense* (5/294, 1.7%), *M. colombiense* (3/294, 1.0%), *M. arosiense* (2/294, 0.7%), and *M. marseillense* (1/294, 0.3%). The results of five single-gene phylogenetic analyses, including 16S rRNA, 23S rRNA, *hsp65*, and *rpoB*, are shown in Table 3 and Fig. S1.

### Comparing the results of MALDI-TOF/MS with phylogenetic analysis

By comparing MALDI-TOF/MS results with concatenated multiple gene phylogenetic analysis (Table 2), only the isolates that were identified as *M. avium* by MALDI-TOF/MS had consistent results. In the other MALDI-TOF/MS group, a variety of species identified through phylogenetic analysis were observed, with *M. intracellulare* clade A accounting for the majority.

**TABLE 2:** Comparison of the differentiation results done by MALDI-TOF/MS and phylogenetic analysis

MALDI-TOF/MS		Phylogenetic analysis	
Species	Number (%)^*a*^	Species	Number (%)^*b*^
*M. intracellulare*	148 (50.3)	*M. intracellulare* clade A	70 (47.3)
		*M*. *intracellulare* subsp. *chimaera*	50 (33.8)
		*M. intracellulare* subsp. *intracellulare*	25 (16.9)
		*M. timonense*	2 (1.4)
		*M. arosiense*	1 (0.7)
*M. chimaera-intracellulare* complex	94 (32.0)	*M. intracellulare* clade A	47 (50.0)
		*M*. *intracellulare* subsp. *chimaera*	33 (35.1)
		*M. intracellulare* subsp. *intracellulare*	13 (13.8)
		*M. arosiense*	1 (1.1)
*M. avium*	35 (11.9)	*M. avium*	35 (100.0)
*M. avium-intracellulare* complex	17 (5.8)	*M. intracellulare* clade A	5 (29.4)
		*M. intracellulare* subsp. *chimaera*	4 (23.5)
		*M. intracellulare* subsp. *intracellulare*	1 (5.9)
		*M. timonense*	3 (17.6)
		*M. colombiense*	3 (17.6)
		*M. marseillense*	1 (5.9)

^*a*^ Percentage of species number accounts for the total 294 isolates.

^*b*^ Percentage of species number accounts for certain species of MALDI-TOF results.

In the phylogenetic analyses based on single genes (16S rRNA, 23S rRNA, *hsp65*, ITS, and *rpoB)*, we found that numerous isolates could not be classified into definite species. The species identification results were also not consistent among these five gene analyses (Table 3).

**TABLE 3:** Comparison of the differentiation results done by full-length 16S rRNA, partial 23S rRNA, partial *hsp65*, partial ITS, and partial *rpoB*

	Concatenated^*a*^	16S rRNA (*n*)	23S rRNA (*n*)	*hsp65* (*n*)	ITS (*n*)	*rpoB* (*n*)
*M. intracellulare* clade A	122	100	0	100	100	17
*M. intracellulare* subsp. *chimaera*	87	111	153	95	106	95
*M. intracellulare* subsp. *intracellulare*	39	40	95	53	42	136
*M. avium*	35	35	37	37	37	37
*M. timonense*	5	4	0	4	4	5
*M. colombiense*	3	2	3	3	3	3
*M. arosiense*	2	0	0	0	0	0
*M. marseillense*	1	1	6	2	2	1
*M. bouchedurhonense*	0	1	0	0	0	0

^*a*^ Full-length 16S rRNA-partial *hsp65*-partial *rpoB.*

### Antimicrobial susceptibility testing

The species differentiation of the 294 isolates used in AST was based on Fig. 1. *M. timonense*, *M. colombiense, M. arosiense, and M. marseillense* were excluded due to a small sample size. The susceptibility patterns of 13 antimicrobial agents of *M. intracellulare* clade A, *M. intracellulare* subsp. *chimaera, M. intracellulare* subsp. *intracellulare*, and *M. avium* are shown in Table 4.

**TABLE 4:** The susceptibility pattern of 13 antimicrobial agents among *Mycobacterium avium* complex (sub)species^*d*^^,^^*e*^

Species/ drug	*M. intracellulare* clade A (*n* = 122)			Susceptibility, *n* (%)^*a*^			*M. intracellulare* subsp. *chimaera* (*n* = 87)			Susceptibility, *n* (%)			*M. intracellulare* subsp. *intracellulare* (*n* = 39)			Susceptibility, *n* (%)			*M. avium* (*n* = 35)			Susceptibility, *n* (%)			
	MIC_50_	MIC_90_	Range	S	I	R	MIC_50_	MIC_90_	Range	S	I	R	MIC_50_	MIC_90_	Range	S	I	R	MIC_50_	MIC_90_	Range	S	I	R	*P* value
AMI^*b*^	16	32	1–64	107 (87.7)	10 (8.2)	5 (4.1)	8	32	1–>64	76 (87.4)	9 (10.3)	2 (2.3)	16	32	1–32	32 (82.1)	7 (17.9)	0 (0)	16	64	8–>64	18 (51.4)	11 (31.4)	6 (17.1)	0.0001
CIP	16	>16	4–>16				16	>16	2–>16				>16	>16	2–>16				16	>16	1–>16				0.0030
CLA^*b*^	2	4	0.25–8	122 (100)	0 (0)	0 (0)	2	2	0.25–64	86 (98.9)	0 (0)	1 (1.1)	2	4	0.5–4	39 (100.0)	0 (0)	0 (0)	2	8	0.12–>64	32 (91.4)	2 (5.7)	1 (2.9)	0.0001
DOX	>16	>16	8–>16				>16	>16	8–>16				>16	>16	16–>16				>16	>16	4–>16				0.0001
EMB	8	16	4–>16				8	16	2–>16				8	32	1–>16				8	>16	4–>16				0.0001
ETH	20	>20	1.2–>20				20	>20	1.2–>20				2.5	>20	0.6–>20				2.5	20	0.6–20				0.0001
INH	8	>8	2–>8				4	>8	0.5–>8				4	>8	0.5–>8				4	>8	2–>8				0.0001
LZD^*b*^	32	64	8–>64	7 (5.7)	24 (19.7)	91 (74.6)	32	32	8–64	3 (3.4)	26 (29.9)	58 (66.7)	32	32	2–64	3 (7.7)	13 (33.3)	23 (59.0)	32	64	8–>64	1 (2.9)	10 (28.6)	24 (68.6)	0.0216
MXF^*b*^	4	4	1–>8	3 (2.5)	45 (36.9)	74 (60.7)	4	8	1–>8	5 (5.7)	23 (26.4)	59 (67.8)	4	8	0.5–8	2 (5.1)	7 (17.9)	30 (76.9)	4	>8	0.5–>8	2 (5.7)	8 (22.9)	25 (71.4)	0.0431
RFB	0.5	1	0.25–2				0.25	1	0.25–4				0.25	1	0.25–4				0.25	1	0.25–4				0.1397
RIF^*c*^	4	8	0.5–>8	1 (0.8)	97 (79.5)	24 (19.7)	4	8	0.5–>8	1 (1.1)	69 (79.3)	17 (19.5)	4	8	1–>8	0 (0)	26 (66.7)	13 (33.3)	8	>8	1–>8	0 (0)	16 (45.7)	19 (54.3)	0.0627
STR	32	64	4–>64				32	64	4–>64				32	64	4–>64				64	>64	16–>64				0.0001
SXT	8/152	>8/152	2/38–>8/152				8/152	>8/152	2/38–>8/152				>8/152	>8/152	2/38–>8/152				8/152	>8/152	0.5/9.5–>8/152				0.4542

^*a*^ S, susceptible; I, intermediate susceptible; and R, resistant.

^*b*^ The breakpoints of AMI, CLA, LZD, and MXF were recommended by the CLSI (20).

^*c*^ The breakpoints of RIF were recommended by van Ingen et al. (21).

^*d*^ MIC_50_: MIC to inhibit the growth of 50% of the isolates; MIC_90_: MIC to inhibit the growth of 90% of the isolates.

^*e*^ The Kruskal-Wallis test was utilized for multiple group comparisons, and *P* values < 0.05 were considered statistically significant.

According to the CLSI methods, CLA showed a high susceptibility rate against *M. intracellulare* clade A (122/122, 100%), *M. intracellulare* subsp. *intracellulare* (39/39, 100%), *M. intracellulare* subsp. *chimaera* (86/87, 98.9%), and *M. avium* (32/35, 91.4%); AMI showed higher susceptibility against *M. intracellulare* clade A (107/122, 87.7%), *M. intracellulare* subsp. *chimaera* (76/87, 87.4%), *M. intracellulare* subsp. *intracellulare* (32/39, 82.1%), but lower susceptibility against *M. avium* (18/35, 51.4%). As for LZD and MXF, the susceptible rates for *M. intracellulare* clade A, *M. intracellulare* subsp. *chimaera, M. intracellulare* subsp. *intracellulare*, and *M. avium* were 5.7% (7/122), 3.4% (3/87), 7.7% (3/39), and 2.9% (1/35) and 2.5% (3/122), 5.7% (5/87), 5.1% (2/39), and 5.7% (2/35), respectively. According to the suggested MIC susceptibility to RIF by van Ingen et al. (21), RIF showed intermediate susceptibility against most *M. intracellulare* clade A (97/122, 79.5%), *M. intracellulare* subsp. *chimaera* (69/87, 79.3%), and *M. intracellulare* subsp. *intracellulare* (26/39, 66.7%); a higher resistance rate against *M. avium* (19/35, 54.3%) was observed.

Out of 13 antimicrobial agents, 10, including AMI, CIP, CLA, DOX, EMB, ETH, INH, LZD, MXF, and STR, exhibited significant differences in susceptibility among these four (sub)species, whereas RFB, RIF, and SXT showed no statistically significant difference in MIC. The MICs of AMI, CLA, EMB, RIF, and STR of *M. avium* were higher than other (sub)species.

**Fig 2:**
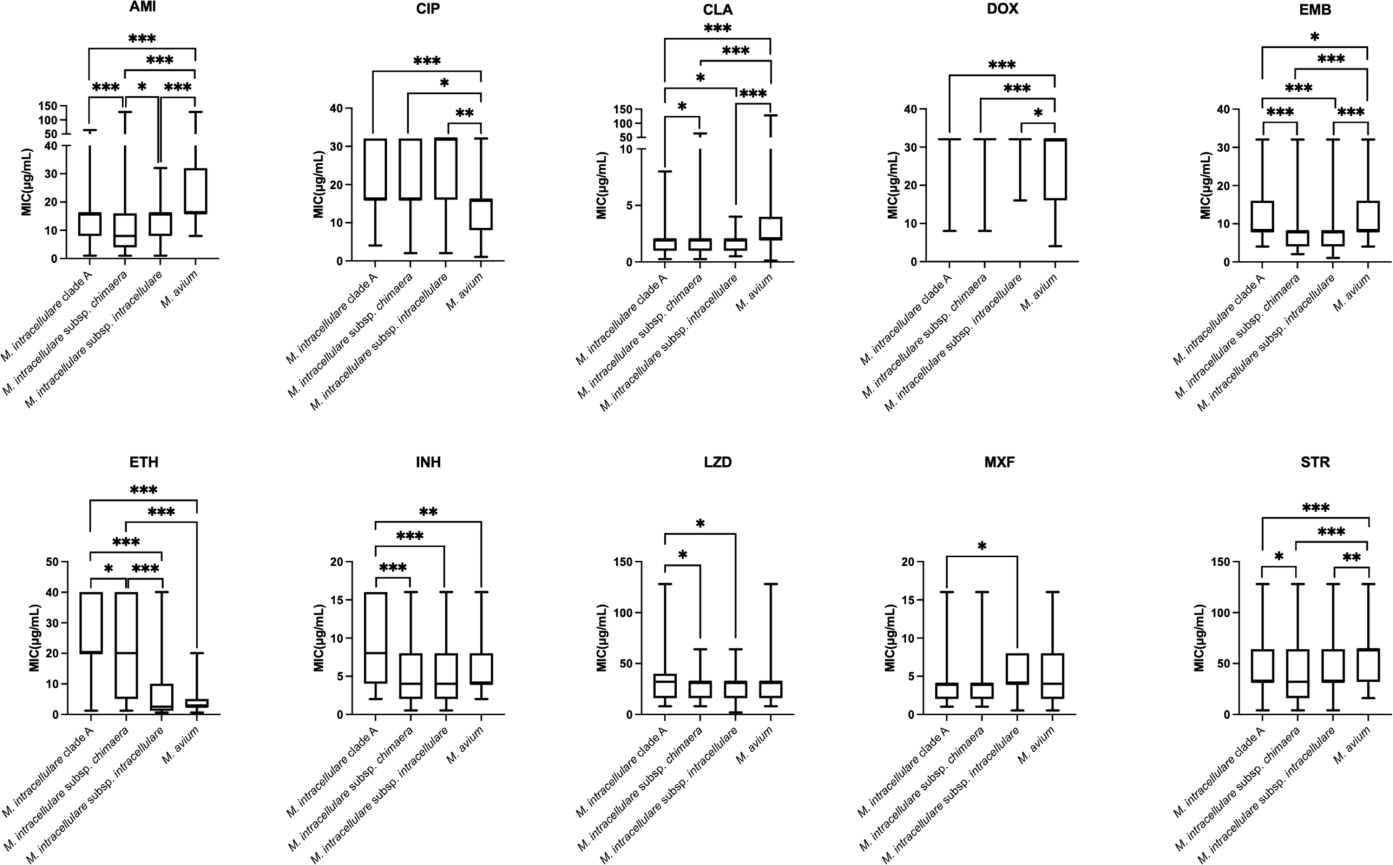
*In vitro* susceptibility of 13 antimicrobial agents among the *Mycobacterium avium* complex (sub)species. GraphPad Prism 9 was used to create statistical graphs for the susceptibility of 13 antimicrobial agents between the species. The MICs of rifampin, rifabutin, and trimethoprim/sulfamethoxazole group comparisons were not included due to the lack of statistical significance based on the Kruskal-Wallis test. A column table was selected to input and organize the MIC data, with each species represented in separate columns. Statistical comparisons between species were performed using the two-sample Mann-Whitney *U* test. Groups showing statistically significant differences were marked on the graph using asterisks. *P < 0.05; **P < 0.01; and ***P < 0.001.

Further susceptibility results between two species among these MAC (sub)species within 13 antimicrobial agents are shown in Fig. 2, which shows numerous significant differences. *M. intracellulare* clade A had higher MICs of ETH and INH compared with *M. intracellulare* subsp. *chimaera* and *M. intracellulare* subsp. *intracellulare*. The MICs of AMI and STR of *M. intracellulare* subsp. *chimaera* were lower than the other three (sub)species. The MICs of AMI, CLA, EMB, and STR of *M. avium* were higher than those of the other three (sub)species. On the other hand, the MICs of CIP and DOX were lower than those of the other three (sub)species.

## DISCUSSION

The importance of identifying each species correctly among MAC is that previous studies have shown that different MAC species are independently associated with diverse risks of disease progression or virulence (22, 23). Compared with using phenotypic features and MALDI-TOF/MS to identify MAC species, WGS is considered the gold standard for species circumscriptions in bacteria. Average nucleotide identity (ANI) and Genome-to-Genome distance (GGD) are viewed as reliable and objective computational techniques for genome comparisons (24). However, the cost of WGS is high and not affordable for all routine clinical laboratories at present. Thus, gene sequencing with phylogenetic analysis is an effective and alternative way to have more accurate species identification of MAC.

In our study, we used concatenated multiple gene sequencing (full-length 16S rRNA-partial *hsp65*-partial *rpoB*) rather than a single gene sequencing to analyze species identification because the concatenated gene analysis has more accurate phylogenies than those inferred from a single gene (25). We found that even the type strains of different species could be classified into the same species in a single gene phylogenetic analysis. Furthermore, the species identification resulting from each gene may vary, which can be confusing and may lead to inappropriate interpretation of the microbiological results.

By using the ANI and GGD methods to classify the genus *Mycobacterium*, *M. yongonense* and *M. chimaera* were reclassified as *M. intracellulare* subsp. *chimaera* (26, 27), which was different from the species identification by van Ingen et al. Thus, the isolates that were classified as *M. intracellulare* subsp. *yongonense* by phylogenetic analysis in Fig. 1 were finally classified as *M. intracellulare* subsp. *chimaera*.

In our study, *M. intracellulare* subsp. *intracellulare* and *M. intracellulare* subsp. *chimaera* isolates can be differentiated by concatenated multi-gene phylogenetic analyses, which were identified as *M. intracellulare*, *M. chimaera-intracellulare* complex, or *M. avium-intracellulare* complex by MALDI-TOF/MS method. As for *M. avium*, the species identification power of MALDI-TOF/MS is consistent with that of phylogenetic analysis. In other words, the distinguishing ability for species identification of the MAC of MALDI-TOF/MS is not inferior to molecular methods for this species. The reason that makes *M. intracellulare* subsp. *intracellulare* and *M. intracellulare* subsp. *chimaera* hard to differentiate by MALDI-TOF/MS is that these two organisms exhibit similar *m/z* patterns. Differentiation is only possible by analyzing their nucleotide differences in 16S rRNA (1 bp) and ITS gene (20 bp) (28, 29). The presence of glycopeptidolipids in the cell wall of *M*. *avium-intracellulare* complex, which prevents protein extraction reagents from penetrating the cell wall, makes these species difficult to identify by MALDI-TOF/MS (28). Thus, differentiation by phylogenetic analysis is more reliable and specific.

A large number of subgroup isolates that had a close relationship with *M. intracellulare* subsp. *intracellulare* were found in this study. However, they were far from the phylogenies of the 18 reference isolates named as *M. intracellulare* clade A, which may indicate intraspecies variation among *M. intracellulare* species in Taiwan.

The anti-mycobacterial regimens against different MAC species have not been established yet, which are all treated by a combination regimen containing a macrolide (CLA or azithromycin), EMB, and a rifamycin (RFB or RIF), suggested by the ATS/ERS/ESCMID/IDSA nontuberculous mycobacterial pulmonary infection treatment guideline (11). *M. intracellulare* clade A, a diverse clonality found in our study, had significantly higher MICs of ETH and INH than the other three (sub)species. It showed a susceptibility pattern similar to that of *M. intracellulare* subsp. *chimaera* and *M. intracellulare* subsp. *intracellulare*, with most isolates being susceptible to AMI and CLA, showing intermediate susceptibility to RIF and higher resistance rate to LZD and MXF. However, *M. intracellulare* clade A exhibited the lowest resistance rate to MXF (60.7%) but the highest to LZD (74.6%) among the four (sub)species. Conversely, *M. intracellulare* subsp. *intracellulare* showed the opposite trend, with the highest resistance rate to MXF (76.9%) and the lowest to LZD (59.0%). This inverse relationship indicates that the choice between moxifloxacin and linezolid as secondary agents for macrolide-resistant MAC isolates or for patients who cannot tolerate macrolide treatment should consider the specific (sub)species involved (30). *M. intracellulare* subsp. *chimaera* exhibited lower MICs of AMI and STR than the other three subspecies. Aminoglycosides may be a good alternative treatment of choice for *M. intracellulare* subsp. *chimaera* pulmonary disease.

AMI had lower susceptibility against *M. avium*, compared with the other three (sub)species. In Fig. 2, *M. avium* had a significant difference in MIC with the other three (sub)species in AMI, CLA, EMB, and STR, as well as the higher MIC. Furthermore, by reviewing the clinical treatment history of patients who yielded *M. avium*, most of them were treatment naive (31/35, 88.6%), while others were cases who had old pulmonary tuberculosis infection history and received anti-TB regimens (RIF, INH, pyrazinamide, and EMB). These results reveal that *M. avium* tends to be a multidrug-resistant species in Taiwan. In other anti-mycobacterial agents, significant differences were also noted between (sub)species, which indicates the treatment of MAC should not be viewed as a single entity.

Our study has several limitations. For the species identification accuracy and further drug resistance gene analysis, WGS plays a crucial role in verifying our study results. However, the cost of the WGS is not affordable in routine laboratories. The isolates we used were only from the single center in Taiwan. The breakpoints used to determine the susceptibility and resistance of 13 antimicrobial agents are not all provided by CLSI. The breakpoints for each MAC have not been established yet. Previous studies have shown that there is no correlation between *in vitro* MIC results and clinical response when using EMB, RIF, and RFB. Therefore, our AST results should be compared with clinical demographics and treatment response in the future.

In conclusion, concatenated gene sequencing (full-length 16S rRNA-partial *hsp65*-partial *rpoB*) with phylogenetic analysis for identifying MAC species differentiation proves to be more accurate and effective compared to MALDI-TOF/MS. Although the international guidelines recommend a combination regimen using a macrolide, EMB, and a rifamycin to treat MAC pulmonary disease, we found interspecies susceptibility variation in our study, which indicated the treatment should be based on species identification and regional susceptibility patterns. *M. avium* tends to be a multidrug-resistant species in Taiwan. Treatment of *M. avium* infection may need additional effective drugs, such as liposomal amikacin, bedaquiline, clofazimine (31), eravacycline, or omadacycline (32). A diverse clonality of *M. intracellulare* was found in our study. To have more appropriate antimicrobial agent options, further clinical features as well as epidemiology should be investigated in the future.

## Data Availability

The representative 882 sequences of the MAC genes analyzed in this study have been deposited in GenBank under the following accession numbers: 16S rRNA (PV602367–PV602660), *hsp65* (PV609108–PV609401), and *rpoB* (PV609402–PV609695).

## SUPPLEMENTAL MATERIAL

Fig. S1 spectrum.00309-25-s0001.docx Phylogenetic analysis of 18 type strains and 294 *Mycobacterium avium* complex isolates based on five single genes.

ASM does not own the copyrights to Supplemental Material that may be linked to, or accessed through, an article. The authors have granted ASM a non-exclusive, world-wide license to publish the Supplemental Material files. Please contact the corresponding author directly for reuse.

## References

[1] Falkinham JO. 2018. Mycobacterium avium complex: adherence as a way of life. AIMS Microbiol 4:428–438. doi:10.3934/microbiol.2018.3.428 31294225 PMC6604937

[2] Ward TT, Rimland D, Kauffman C, Huycke M, Evans TG, Heifets L. 1998. Randomized, open-label trial of azithromycin plus ethambutol vs. clarithromycin plus ethambutol as therapy for Mycobacterium avium complex bacteremia in patients with human immunodeficiency virus infection. Veterans Affairs HIV Research Consortium. Clin Infect Dis 27:1278–1285. doi:10.1086/514999 9827282

[3] Ye JJ, Wu TS, Chiang PC, Lee MH. 2007. Factors that affect sputum conversion and treatment outcome in patients with Mycobacterium avium-intracellulare complex pulmonary disease. J Microbiol Immunol Infect 40:342–348. 17712469

[4] van Ingen J, Turenne CY, Tortoli E, Wallace RJ Jr, Brown-Elliott BA. 2018. A definition of the Mycobacterium avium complex for taxonomical and clinical purposes, a review. Int J Syst Evol Microbiol 68:3666–3677. doi:10.1099/ijsem.0.003026 30231956

[5] Boyle DP, Zembower TR, Qi C. 2015. Evaluation of Vitek MS for rapid classification of clinical isolates belonging to Mycobacterium avium complex. Diagn Microbiol Infect Dis 81:41–43. doi:10.1016/j.diagmicrobio.2014.09.026 25445119

[6] Kim SH, Shin JH. 2018. Identification of nontuberculous mycobacteria using multilocous sequence analysis of 16S rRNA, hsp65, and rpoB. J Clin Lab Anal 32:e22184. doi:10.1002/jcla.22184 28230286 PMC6817141

[7] Ben Salah I, Adékambi T, Raoult D, Drancourt M. 2008. rpoB sequence-based identification of Mycobacterium avium complex species. Microbiology (Reading) 154:3715–3723. doi:10.1099/mic.0.2008/020164-0 19047739

[8] Chawla R, Shaw B, von Bredow B, Chong C, Garner OB, Zangwill KM, Yang S. 2023. Accurate subspecies-level identification of clinically significant Mycobacterium avium and Mycobacterium intracellulare by whole-genome sequencing. J Microbiol Methods 208:106726. doi:10.1016/j.mimet.2023.106726 37120137

[9] de Zwaan R, van Ingen J, van Soolingen D. 2014. Utility of rpoB gene sequencing for identification of nontuberculous mycobacteria in the Netherlands. J Clin Microbiol 52:2544–2551. doi:10.1128/JCM.00233-14 24808238 PMC4097680

[10] Park H, Jang H, Kim C, Chung B, Chang CL, Park SK, Song S. 2000. Detection and identification of mycobacteria by amplification of the internal transcribed spacer regions with genus- and species-specific PCR primers. J Clin Microbiol 38:4080–4085. doi:10.1128/JCM.38.11.4080-4085.2000 11060072 PMC87545

[11] Daley CL, Iaccarino JM, Lange C, Cambau E, Wallace RJ Jr, Andrejak C, Böttger EC, Brozek J, Griffith DE, Guglielmetti L, Huitt GA, Knight SL, Leitman P, Marras TK, Olivier KN, Santin M, Stout JE, Tortoli E, van Ingen J, Wagner D, Winthrop KL. 2020. Treatment of nontuberculous mycobacterial pulmonary disease: an official ATS/ERS/ESCMID/IDSA clinical practice guideline. Clin Infect Dis 71:e1–e36. doi:10.1093/cid/ciaa241 32628747 PMC7768748

[12] Zelazny AM, Root JM, Shea YR, Colombo RE, Shamputa IC, Stock F, Conlan S, McNulty S, Brown-Elliott BA, Wallace RJ Jr, Olivier KN, Holland SM, Sampaio EP. 2009. Cohort study of molecular identification and typing of Mycobacterium abscessus, Mycobacterium massiliense, and Mycobacterium bolletii. J Clin Microbiol 47:1985–1995. doi:10.1128/JCM.01688-08 19420162 PMC2708513

[13] Jong BE, Wu TS, Chen NY, Yang CH, Shu CC, Wang LS, Wu TL, Lu JJ, Chiu CH, Lai HC, Chung WH. 2022. Impact on macrolide resistance of genetic diversity of Mycobacterium abscessus species. Microbiol Spectr 10:e0274922. doi:10.1128/spectrum.02749-22 36416559 PMC9769998

[14] Edwards U, Rogall T, Blöcker H, Emde M, Böttger EC. 1989. Isolation and direct complete nucleotide determination of entire genes. Characterization of a gene coding for 16S ribosomal RNA. Nucleic Acids Res 17:7843–7853. doi:10.1093/nar/17.19.7843 2798131 PMC334891

[15] Meier A, Kirschner P, Springer B, Steingrube VA, Brown BA, Wallace RJ Jr, Böttger EC. 1994. Identification of mutations in 23S rRNA gene of clarithromycin-resistant Mycobacterium intracellulare. Antimicrob Agents Chemother 38:381–384. doi:10.1128/AAC.38.2.381 8192472 PMC284463

[16] Telenti A, Marchesi F, Balz M, Bally F, Böttger EC, Bodmer T. 1993. Rapid identification of mycobacteria to the species level by polymerase chain reaction and restriction enzyme analysis. J Clin Microbiol 31:175–178. doi:10.1128/jcm.31.2.175-178.1993 8381805 PMC262730

[17] Harmsen D, Dostal S, Roth A, Niemann S, Rothgänger J, Sammeth M, Albert J, Frosch M, Richter E. 2003. RIDOM: comprehensive and public sequence database for identification of Mycobacterium species. BMC Infect Dis 3:26. doi:10.1186/1471-2334-3-26 14611664 PMC280682

[18] Tamura K, Stecher G, Kumar S. 2021. MEGA11: molecular evolutionary genetics analysis version 11. Mol Biol Evol 38:3022–3027. doi:10.1093/molbev/msab120 33892491 PMC8233496

[19] CLSI. 2003. Susceptibility testing of Nocardia, and other aerobic actinomycetes; approved standard- second edition. The Clinical and Laboratory Standards Institute, Wayne, PA. 31339680

[20] CLSI. 2018. Performance standards for susceptibility testing of mycobacteria, Norcardia spp., and other aerobic actinomycetes. Clinical and Laboratory Standards Institute. 31339680

[21] van Ingen J, Egelund EF, Levin A, Totten SE, Boeree MJ, Mouton JW, Aarnoutse RE, Heifets LB, Peloquin CA, Daley CL. 2012. The pharmacokinetics and pharmacodynamics of pulmonary Mycobacterium avium complex disease treatment. Am J Respir Crit Care Med 186:559–565. doi:10.1164/rccm.201204-0682OC 22744719

[22] Pan SW, Shu CC, Feng JY, Chien JY, Wang JY, Chan YJ, Yu CJ, Su WJ. 2021. Impact of different subspecies on disease progression in initially untreated patients with Mycobacterium avium complex lung disease. Clin Microbiol Infect 27:467. doi:10.1016/j.cmi.2020.04.020 32360207

[23] Stout JE, Koh WJ, Yew WW. 2016. Update on pulmonary disease due to non-tuberculous mycobacteria. Int J Infect Dis 45:123–134. doi:10.1016/j.ijid.2016.03.006 26976549

[24] Tortoli E, Meehan CJ, Grottola A, Fregni Serpini G, Fabio A, Trovato A, Pecorari M, Cirillo DM. 2019. Genome-based taxonomic revision detects a number of synonymous taxa in the genus Mycobacterium. Infect Genet Evol 75:103983. doi:10.1016/j.meegid.2019.103983 31352146

[25] Gadagkar SR, Rosenberg MS, Kumar S. 2005. Inferring species phylogenies from multiple genes: concatenated sequence tree versus consensus gene tree. J Exp Zool Pt B 304B:64–74. doi:10.1002/jez.b.21026 15593277

[26] Castejon M, Menéndez MC, Comas I, Vicente A, Garcia MJ. 2018. Whole-genome sequence analysis of the Mycobacterium avium complex and proposal of the transfer of Mycobacterium yongonense to Mycobacterium intracellulare subsp. yongonense subsp. nov. Int J Syst Evol Microbiol 68:1998–2005. doi:10.1099/ijsem.0.002767 29683417

[27] Nouioui I, Brunet LR, Simpson D, Klenk HP, Goodfellow M. 2018. Description of a novel species of fast growing mycobacterium: Mycobacterium kyogaense sp. nov., a scotochromogenic strain received as Mycobacterium vaccae. Int J Syst Evol Microbiol 68:3726–3734. doi:10.1099/ijsem.0.003039 30300123

[28] Mareković I, Bošnjak Z, Jakopović M, Boras Z, Janković M, Popović-Grle S. 2016. Evaluation of matrix-assisted laser desorption/ionization time-of-flight mass spectrometry in identification of nontuberculous mycobacteria. Chemotherapy 61:167–170. doi:10.1159/000442517 26821270

[29] Tortoli E, Rindi L, Garcia MJ, Chiaradonna P, Dei R, Garzelli C, Kroppenstedt RM, Lari N, Mattei R, Mariottini A, Mazzarelli G, Murcia MI, Nanetti A, Piccoli P, Scarparo C. 2004. Proposal to elevate the genetic variant MAC-A, included in the Mycobacterium avium complex, to species rank as Mycobacterium chimaera sp. nov. Int J Syst Evol Microbiol 54:1277–1285. doi:10.1099/ijs.0.02777-0 15280303

[30] CLSI. 2011. Susceptibility testing of mycobacteria, Nocardiae, and other aerobic Actinomycetess; approved standard—second edition. Clinical and Laboratoy Standards Institute, Wayne, PA, USA. 31339680

[31] van Ingen J, Obradovic M, Hassan M, Lesher B, Hart E, Chatterjee A, Daley CL. 2021. Nontuberculous mycobacterial lung disease caused by Mycobacterium avium complex - disease burden, unmet needs, and advances in treatment developments. Expert Rev Respir Med 15:1387–1401. doi:10.1080/17476348.2021.1987891 34612115

[32] Kaushik A, Ammerman NC, Martins O, Parrish NM, Nuermberger EL. 2019. In vitro activity of new tetracycline analogs omadacycline and eravacycline against drug-resistant clinical isolates of Mycobacterium abscessus. Antimicrob Agents Chemother 63:e00470-19. doi:10.1128/AAC.00470-19 30962331 PMC6535573

