# Supplementary material for: Heterogeneity among Mycobacterium avium complex species isolated from pulmonary infection in Taiwan
Source: Microbiol Spectr. 2025 Jul 7;13(8):e00309-25. doi: 10.1128/spectrum.00309-25 (PMC12323620; doi:10.1128/spectrum.00309-25)

Figure S1. Phylogenetic analysis of 18 type strains and 294 *Mycobacterium avium* complex isolates based on five single genes. A. 16S rRNA, B. 23S rRNA, C. *hsp65*, D. ITS, E. *rpoB.*

A.
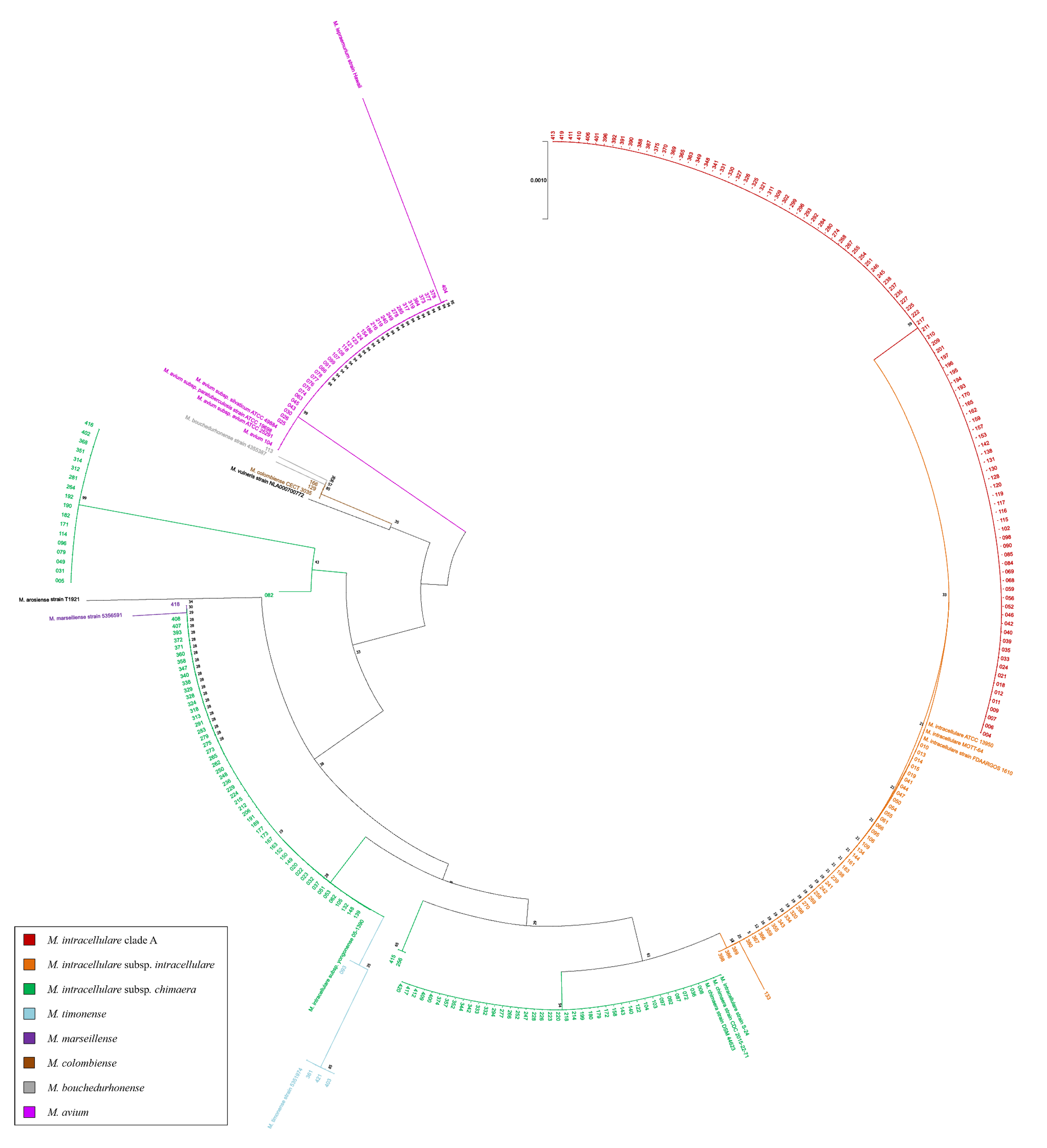


B.
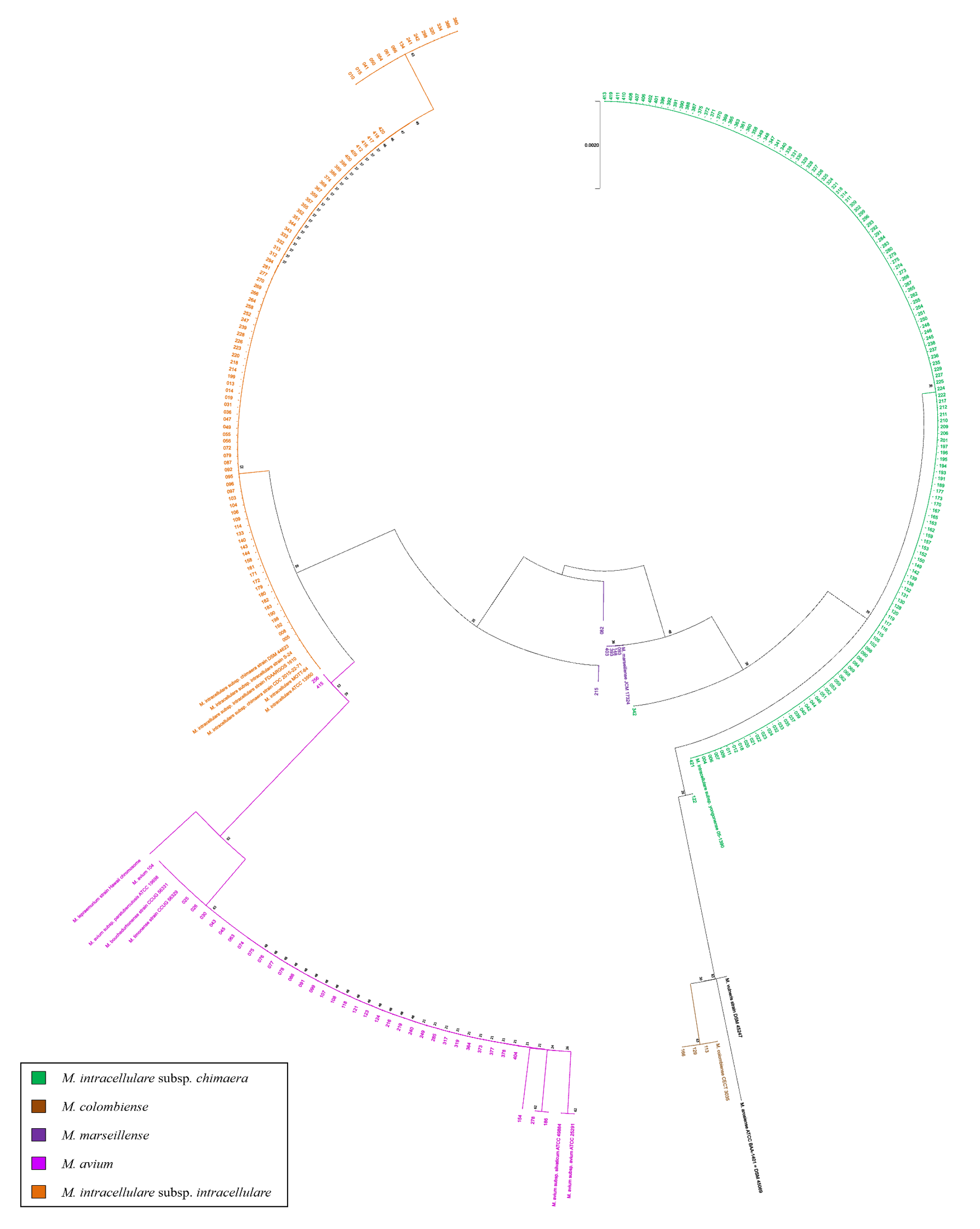


C.


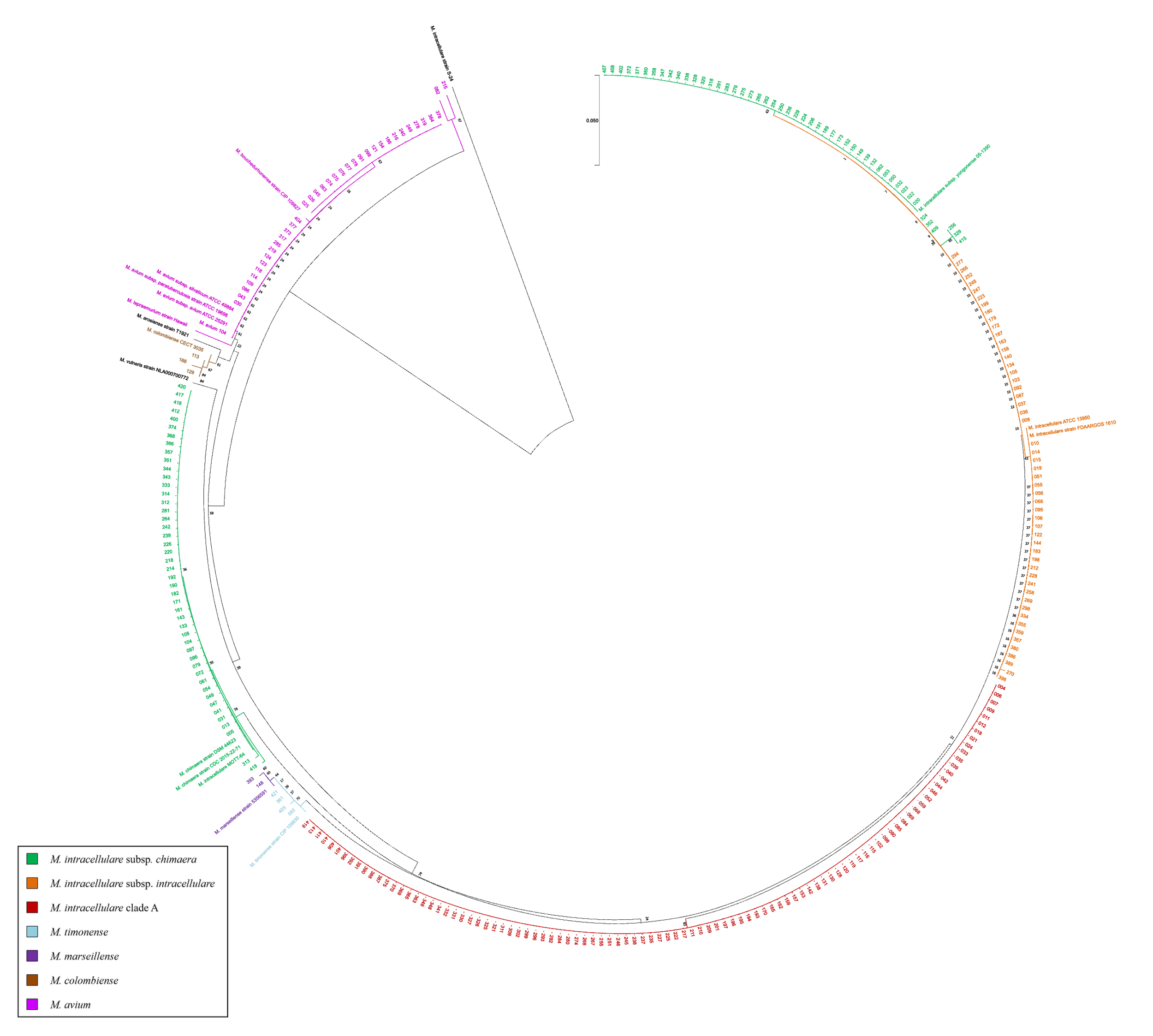


D.
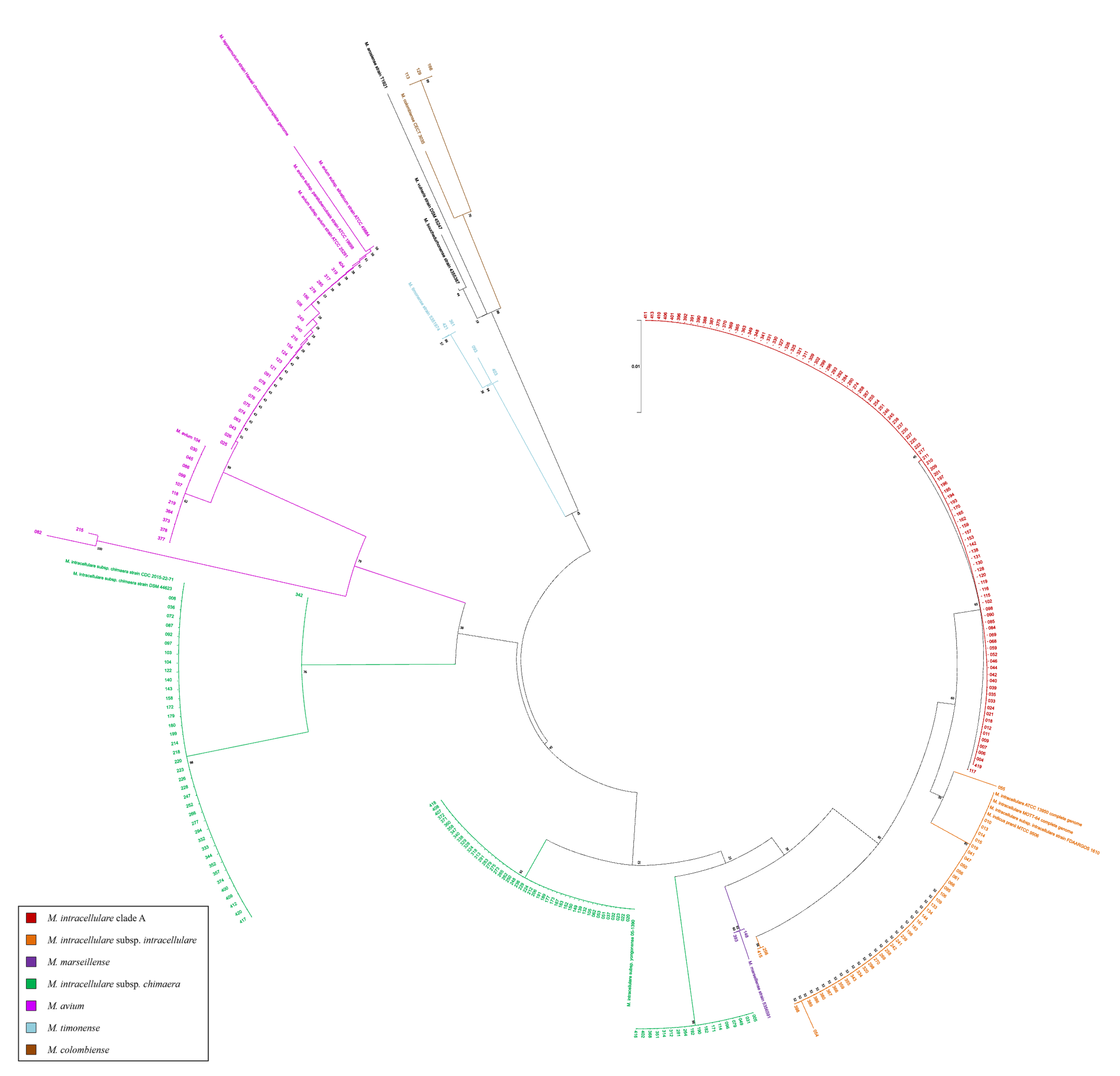


E.


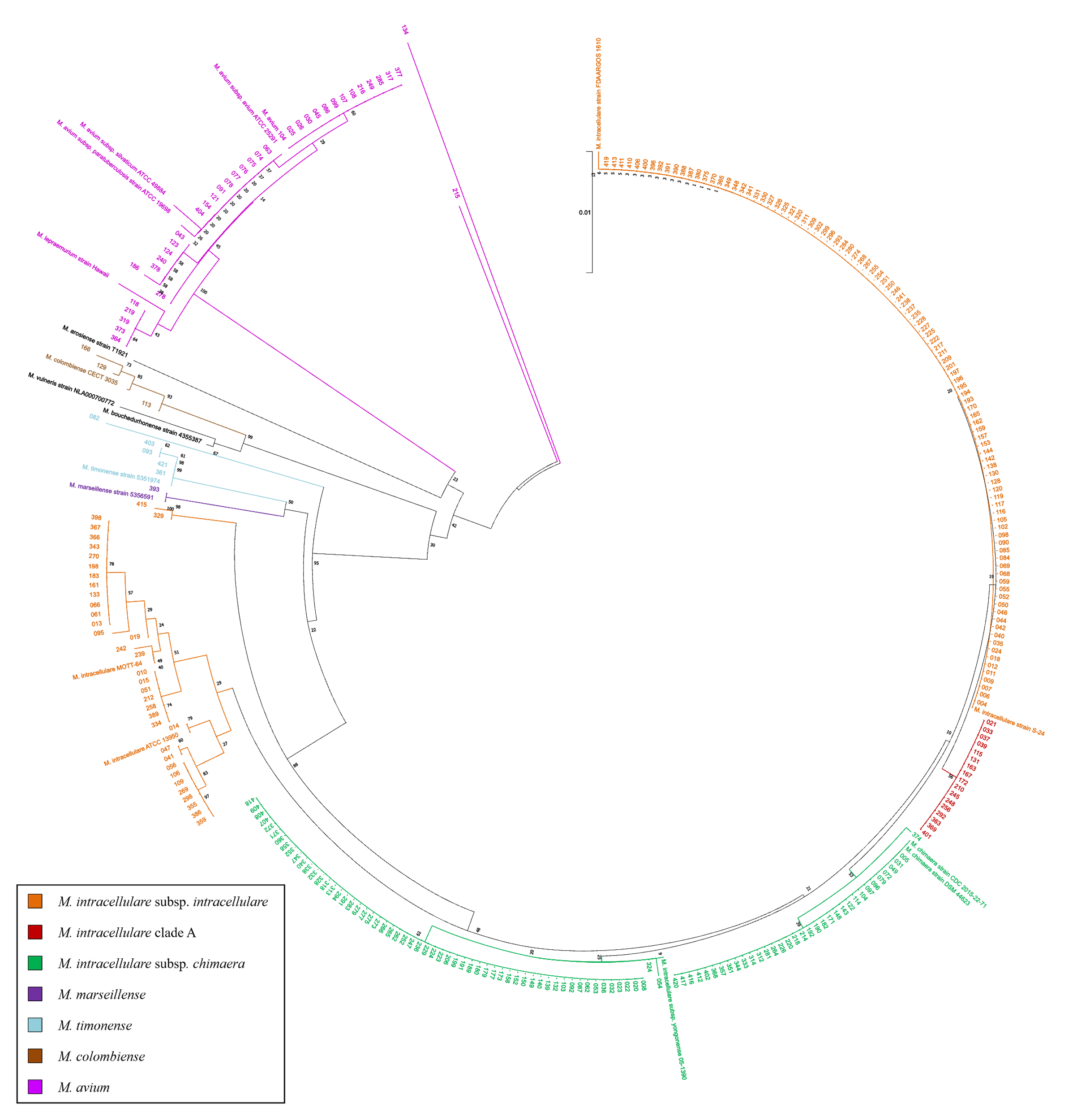

Supplement: Fig. S1 — Phylogenetic analysis of 18 type strains and 294 Mycobacterium avium complex isolates based on five single genes. [file spectrum.00309-25-s0001.docx]
